# Innovative Technique for Single-Stage Phalloplasty with Complete Urethral Reconstruction for Micropenis and Perineal Hypospadias

**DOI:** 10.1055/s-0045-1812104

**Published:** 2025-12-30

**Authors:** Mohit Sharma, Vasundhra Jain, Srilekha Reddy Galigutta, Shikha Gupta, Georgie Mathew, Devajyoti Guin, Anil Murarka

**Affiliations:** 1 Department of Plastic and Reconstructive Surgery, Amrita Hospital, Faridabad, Haryana, India; 2 Department of Urology, Amrita Institute of Medical Sciences, Kochi, Kerala, India

**Keywords:** urethral reconstruction, penile reconstruction, hypospadias

## Abstract

In a 20-year-old male patient born with micropenis and urethral opening in the perineal region, total phallus and bulbar portions of the urethra were reconstructed in a single stage using the radial artery forearm flap. The penile reconstruction followed a tube-in-tube design, and for the bulbar urethral reconstruction, an additional length of radial artery forearm flap was designed and reinforced with deep fascia of the forearm. Gracilis muscle was harvested from the opposite thigh and used to reinforce the urethral anastomotic site. The descending branch of the lateral circumflex femoral artery was chosen to vascularize the reconstructed urethra and penis, using this vessel as the recipient, giving a distinct advantage over the deep inferior epigastric or femoral vessels. To the best of our knowledge, this technique of total penile and urethral reconstruction in a single stage has not been described in the literature, and this would be a useful technique in the armamentarium of penile reconstruction.

## Introduction

Micropenis with perineal hypospadias is a crippling congenital anomaly with profound psychological and physical consequences for the patient who suffers from it. With no possibility of normal sexual intercourse and the need to sit down in a squatting position for micturition, it is quite devastating for a young adult and otherwise healthy male.

The goal of total phallic reconstruction is the creation of a sensate and cosmetically acceptable phallus of adequate length that allows the patient to void in a standing position and engage in penetrative sexual intercourse with confidence. 1

## Case Report

**Fig. 1:**
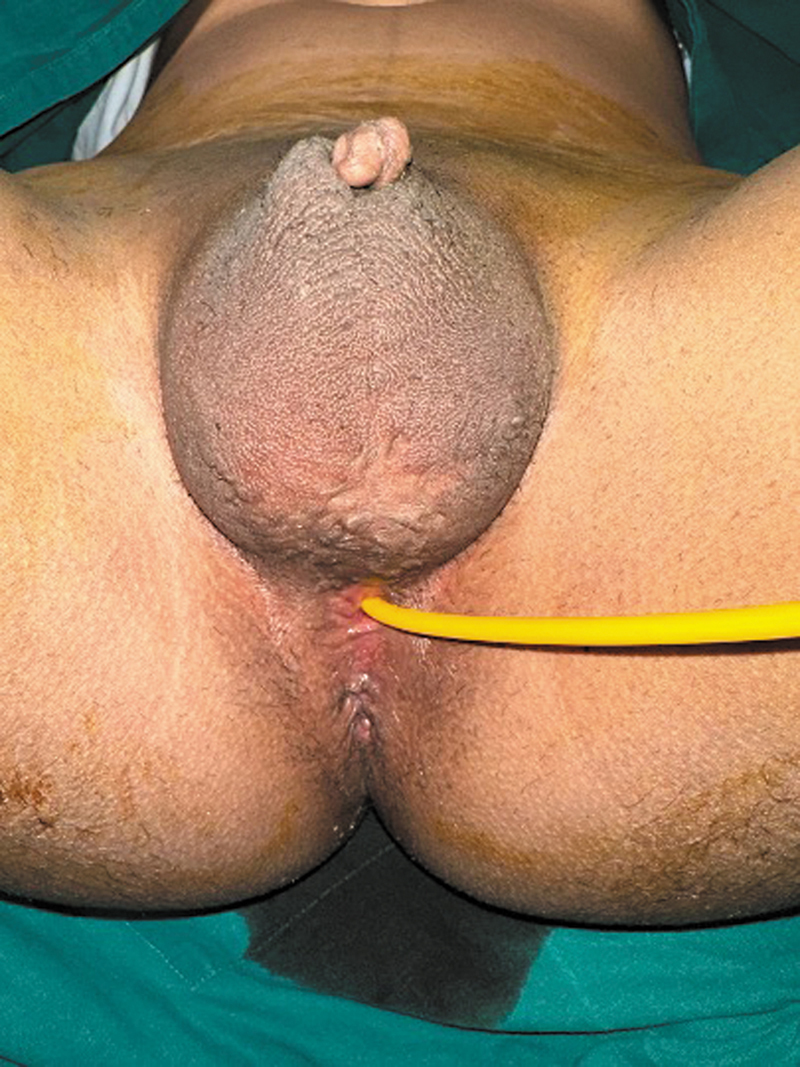
Preoperative photo demonstrating patient with micropenis and perineal urethra.

Our patient was a 20-year-old male suffering from micropenis with perineal hypospadias ( Fig. 1 ). He was planned for single-stage reconstruction of the total phallus and bulbar portions of the urethra with a radial forearm free flap.

In the consultation room, he underwent Allen's test to establish the nondominance of the radial circulation. He received nine sessions of Nd:YAG 1064 laser for hair reduction from the prospective urethral site over a period of 9 months.

### Preoperative Investigations

Preoperative urethrogram was done to determine the end of the urethral remnant.

**Fig. 2:**
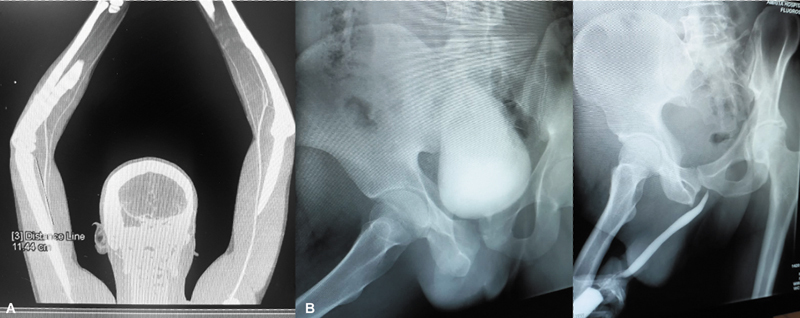
( **A** ) Preoperative CT angiography of the donor limb demonstrating the aberrant take-off of the radial artery. ( **B** ) Preoperative MCU and RGU. CT, computed tomography; MCU, micturating cystourethrogram; RGU, retrograde urethrogram.

He underwent computed tomography angiography of his nondominant left upper limb. The patient had an aberrant origin of radial artery from the mid-brachial artery ( Fig. 2 ).

### Surgical Technique

#### Flap Design

**Fig. 3:**
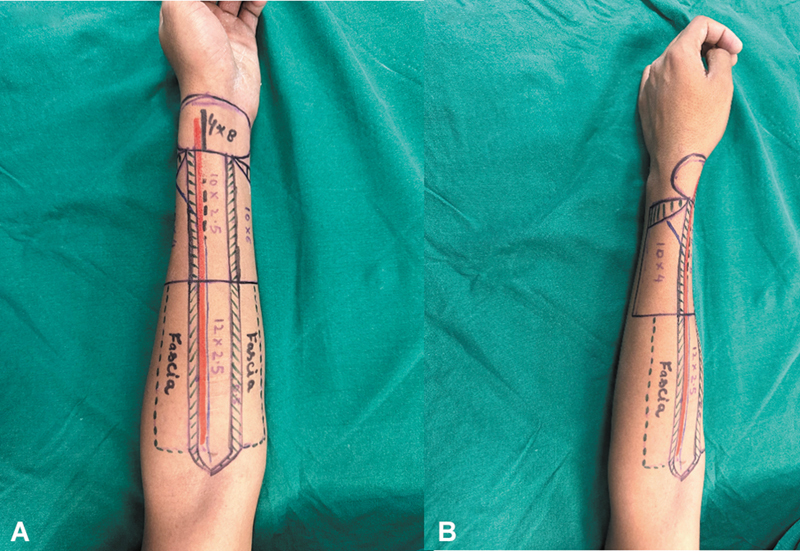
( **A** ) Ventral view of the flap design demonstrating a long segment of urethra of 22 cm centered over the radial artery with marking of the neophallus. ( **B** ) Lateral view of the flap marking.

The radial forearm flap design consists of three components—neourethra, shaft of the phallus, and the glans. The neourethra was designed with a diameter of 2.5 cm, giving a total urethral circumference of 2 × 3.14 × 1.25 = 7.85 cm, and 22 cm in length, which was centered on the radial artery ( Fig. 3 ). 2

#### Flap Dissection and Phallus Creation

The phallic and urethral reconstruction surgery was divided into two stages:

Stage I: The flap was raised in the subfascial plane and the radial artery along with venae comitantes was dissected out. The excess length of the urethra was reinforced with deep fascia of the forearm. Foley's catheter of 16 French was placed over the prospective urethra.

The urethral tube creation was done in three layers using 3–0 PDS. The lateral and medial wings of the radial forearm flap were sutured to each other with 3/0 PDS continuous suture to complete the penile and urethral construct.

**Fig. 4:**
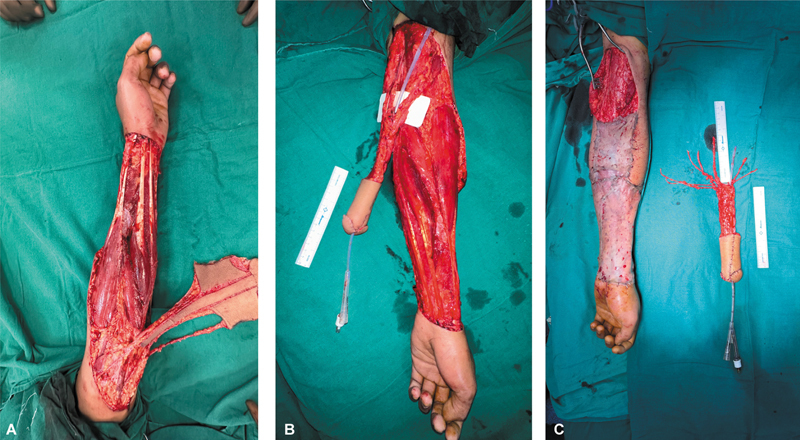
( **A** ) Intraoperative view of the flap harvest with de-epithelialized portion along with the radial artery pedicle and cephalic vein. ( **B** ) Intraoperative picture demonstrating the phallus creation while the phallus is still attached to the radial artery pedicle. The phallus creation was done with the Foley catheter in situ. ( **C** ) Flap harvested along with the radial artery pedicle and cephalic vein, and medial and lateral antebrachial cutaneous nerves, with SSG placed over the donor site. SSG, split skin graft.

We wanted to be absolutely sure about the blood supply of the shaft and urethral skin; therefore, we left the new penile and urethral construct on the forearm for 48 hours and covered the raw area with a skin graft ( Fig. 4 ).

#### Local Dissection

**Video 1 (A, B)** Surgical technique demonstrating single-stage reconstruction with postoperative voiding.




**Fig. 5:**
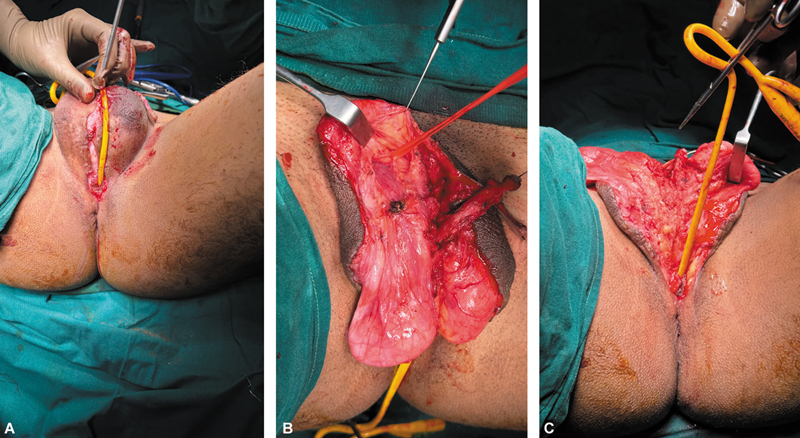
( **A** ) Intraoperative view of the perineal urethra with Foley catheter in situ. ( **B** ) Intraoperative view demonstrating the degloved micropenis and the ilioinguinal nerve. ( **C** ) Native urethral opening after the dissection was completed.

The micropenis was degloved and the skin, corpora cavernosa, and spongiosum containing the urethra are separated. The scrotum was split at the midline raphe to expose the urethra. The dorsal nerve of the penis was of small size as a direct consequence of the small penile shaft; hence, the ilioinguinal nerve was also dissected and isolated on the right groin region so as to provide adequate sensory input for the tactile sensation ( Fig. 5 ).

Stage II: This was done on the 3rd postoperative day (POD). The flap was harvested and transferred to the perineum, where the urethral anastomosis was performed in a single layer using 3–0 PDS sutures. We decided to harvest contralateral gracilis muscle which was then transferred subcutaneously to the perineum and used to reinforce the urethral anastomosis to further reduce any chance of urinary leak. The arterial and venous anastomoses were performed in the right groin using 9–0 Nylon interrupted sutures. The lateral antebrachial cutaneous nerve was anastomosed to the ilioinguinal nerve and the medial antebrachial nerve coaptation was done to the dorsal nerve of penis using 10–0 Nylon and tissue glue ( Video 1[A , B] ).

### Recipient Vessels

The descending branch of the lateral circumflex femoral artery on the right side was used as the recipient pedicle and the great saphenous vein for additional venous drainage.

### Postoperative

**Video 2** Postoperative videoscopy.


Patient's catheter was removed on POD-21 and he was allowed to micturate in the standing position. Good urinary stream was noted and no leak was detected.

He did not have any complications postoperatively. He is being followed up regularly and he has gained sensation in the neophallus till the tip of the glans.

**Fig. 6:**
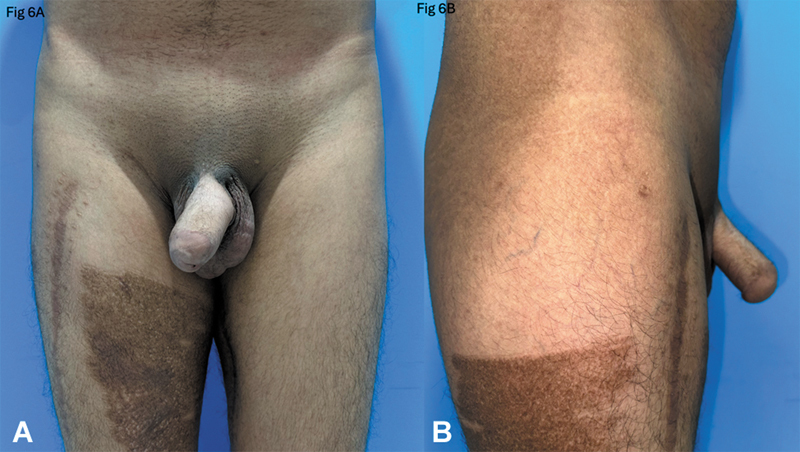
( **A** ) Postoperative result at 1 year—frontal view. ( **B** ) Postoperative result at 1 year—side view.

At 1 year, his urodynamic study had an average urinary flow rate of 11 mL/s and a maximum flow rate of 20 mL/s, which closely conforms to the normal adult flow rate during micturition. 3 On videoscopy, he was found to have no strictures and minimal hair growth in the neourethra ( Fig. 6 , Video 2 ).

The patient is awaiting insertion of a penile stiffener implant to permit sexual function.

## Discussion

In the literature, to the best of our knowledge, we have not come across any other case report or series demonstrating simultaneous long-segment urethral reconstruction and phallus reconstruction using a single free radial forearm flap.

Total reconstruction of the phallus with simultaneous reconstruction of a long segment of urethra is a challenge. Previously, cases have been reported where an expanded sensate lateral forearm flap has been performed in a patient in the case of traumatic penile amputation. 4

We decided to use a single radial artery forearm flap (RAFF) for the reconstruction with design modification to incorporate a longer urethral segment of approximately 22 cm. As a longer flap was harvested to incorporate the urethral length at the time of planning, we decided to use vein grafts for anastomosis. But in this case due to the presence of aberrant radial artery origin from the mid-brachial artery, the need for vein graft was avoided.

In our patient, we achieved a phallus length of 10 cm, which is adequate for sexual intercourse and for voiding in the standing position.

## Conclusion

The simultaneous reconstruction of the total penis with complete bulbar urethra with total urethral length of 22 cm has never been described in the literature to the best of our knowledge. This novel concept has the potential to become the standard of care for such complex hypospadias and micropenis cases.
